# Using Dental Pulp Stem Cells for Stroke Therapy

**DOI:** 10.3389/fneur.2019.00422

**Published:** 2019-04-29

**Authors:** Maria R. Gancheva, Karlea L. Kremer, Stan Gronthos, Simon A. Koblar

**Affiliations:** ^1^Stroke Research Programme Laboratory, Adelaide Medical School, Faculty of Health and Medical Sciences, University of Adelaide, Adelaide, SA, Australia; ^2^Mesenchymal Stem Cell Laboratory, Adelaide Medical School, Faculty of Health and Medical Sciences, University of Adelaide, Adelaide, SA, Australia; ^3^South Australian Health and Medical Research Institute, Adelaide, SA, Australia; ^4^Central Adelaide Local Health Network, Adelaide, SA, Australia

**Keywords:** cell-based therapy, ischaemic stroke, dental pulp stem cells, neural stem cells, multipotent, reprogramming, differentiation, neural crest

## Abstract

Stroke is a leading cause of permanent disability world-wide, but aside from rehabilitation, there is currently no clinically-proven pharmaceutical or biological agent to improve neurological disability. Cell-based therapies using stem cells, such as dental pulp stem cells, are a promising alternative for treatment of neurological diseases, including stroke. The ischaemic environment in stroke affects multiple cell populations, thus stem cells, which act through cellular and molecular mechanisms, are promising candidates. The most common stem cell population studied in the neurological setting has been mesenchymal stem cells due to their accessibility. However, it is believed that neural stem cells, the resident stem cell of the adult brain, would be most appropriate for brain repair. Using reprogramming strategies, alternative sources of neural stem and progenitor cells have been explored. We postulate that a cell of closer origin to the neural lineage would be a promising candidate for reprogramming and modification towards a neural stem or progenitor cell. One such candidate population is dental pulp stem cells, which reside in the root canal of teeth. This review will focus on the neural potential of dental pulp stem cells and their investigations in the stroke setting to date, and include an overview on the use of different sources of neural stem cells in preclinical studies and clinical trials of stroke.

## Introduction

The central nervous system (CNS) functions through complex molecular and cellular interactions, and disruption by severe injury or disease leads to irreversible neuronal loss and associated functional deficits. This results in highly debilitating pathologies associated with significant health and economic burden for patients, their families, carers, and the health systems.

Stroke is a global health care problem and a leading cause of acquired adult neurological disability (1). With an aging population, the incidence and prevalence of stroke is predicted to rise. A stroke is characterised by reduced and insufficient blood supply to part of the brain. Inadequate oxygen and nutrients lead to tissue infarction, resulting in disability due to loss-of-function associated with the damaged area of the brain.

There are two main types of stroke; haemorrhagic and ischaemic. Haemorrhagic strokes, accounting for 13 percent of strokes (1), result from bleeding when a blood vessel is ruptured. Ischaemic stroke is the most common presentation of stroke at 87 percent of all cases (1), and is due to an obstruction in the blood supply, which could be formed locally (thrombosis) or formed elsewhere in the body (embolism).

During an ischaemic stroke, a complex chain of events takes place at the molecular and cellular levels, which results in cell necrosis at the site of the vascular insult (the ischaemic core), while the region surrounding the core (the ischaemic penumbra) remains viable for some time due to collateral blood supply and can thus be salvaged. A strong inflammatory response is initiated within hours of stroke onset, characterised by reactive astrogliosis, microglial activation, disruption to the blood-brain barrier (BBB), and infiltration of neutrophils and monocytes/macrophages (2). Growth factors and inflammatory mediators, from local glial and inflammatory cells, alter the reaction of endogenous neural stem and progenitor cells. Over time, reorganisation of the neural network around the core takes place. If untreated, the penumbra will transform into ischaemic tissue, expanding the irreversibly damaged area of brain. There is an opportunity to save the penumbral tissue via acute recanalisation therapies.

The currently available therapeutic interventions, such as thrombectomy and thrombolysis, are limited to a narrow therapeutic window and eligibility criteria, and though they have a significant impact on stroke outcome, disability remains after any intervention. Thrombectomy refers to the mechanical removal of a blood clot, which has been effective when performed within 24 h post-stroke (3). The more common intervention is thrombolysis by intravenously administered recombinant tissue plasminogen activator, to breakdown the clot. This is currently the only approved pharmacological agent that shows significant benefits in acute ischaemic stroke, but is only applicable within a short time frame of 4.5 h from symptomatic onset (4). Unfortunately, many patients are ineligible for these reperfusion therapies. In addition, poor patient outcomes can still be observed. Once a stroke patient is stabilised, rehabilitation interventions are relied upon to promote neuroplasticity, as patients adapt to residual disability. Improvements are most significant in the first several months following a stroke (5). There is currently no therapy that can restore damaged neural tissue and its associated functions.

Cell-based therapies have the potential to promote functional recovery in patients affected by stroke and other neurological diseases. Stem cells are promising candidates, as they can act through multiple cellular and molecular mechanisms to provide support for endogenous cells, stimulate endogenous processes, and act as a source of cell replacement. Neural stem cells (NSC), which reside in specific areas of the CNS, are the most appropriate stem cells for brain repair. Research is focused on two therapeutic paradigms; enhancing and manipulating endogenous NSC, and implanting exogenous NSC. Reprogramming strategies are being applied to develop NSC from more easily accessible and abundant cell types (6). Dental pulp stem cells (DPSC) are adult stem cells obtained from the dental pulp tissue in the tooth chamber (7). These cells are easily sourced and have neurogenic potential. They are being investigated as an alternative source of neural cells and in preclinical models of neurological diseases, including stroke. This review will focus on the potential use of human DPSC for stroke therapy and will include an overview of different types of NSC being studied.

## Types of Stem Cells

**Figure 1:**
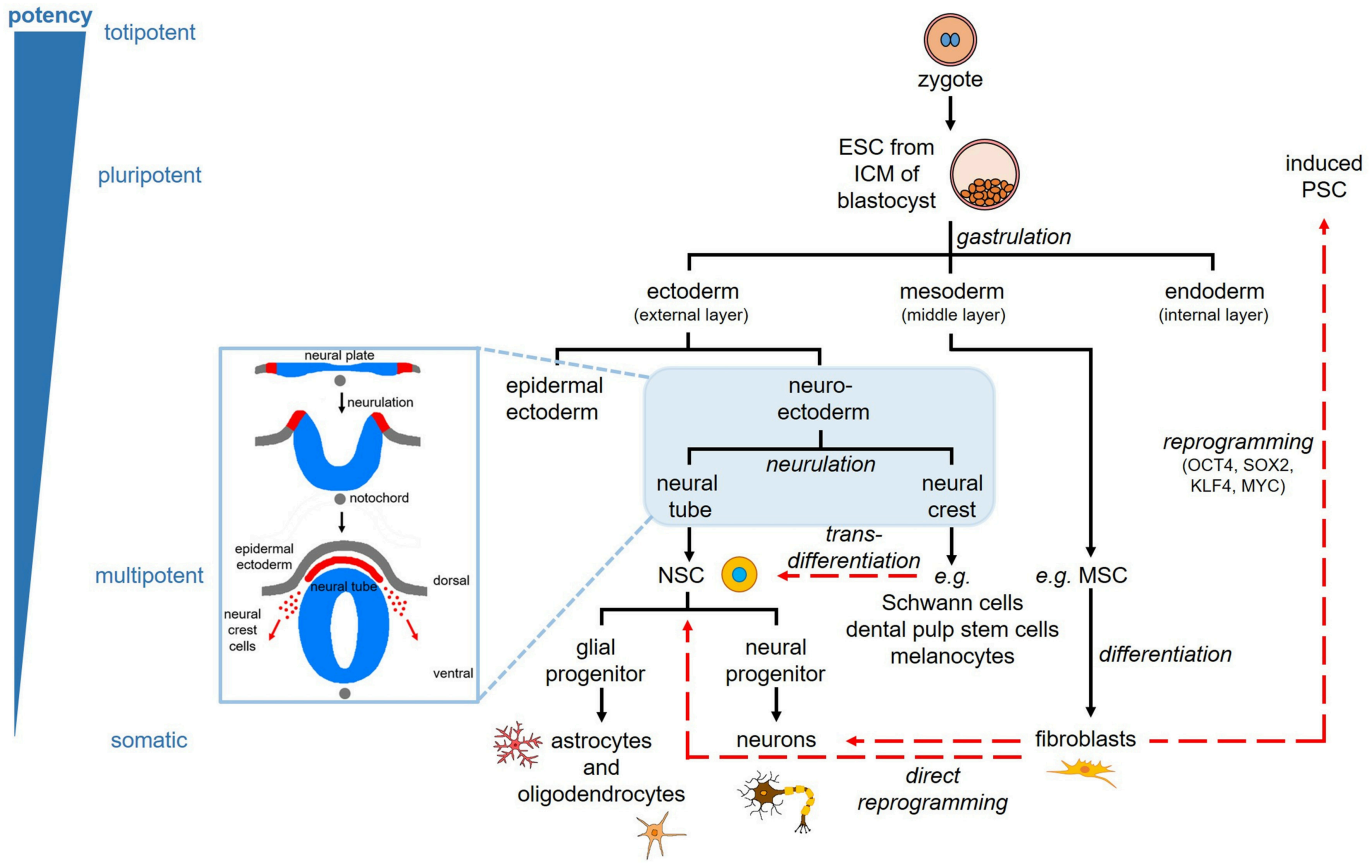
Stem cell lineage and reprogramming. Pluripotent embryonic stem cells (ESC) are derived from the inner cell mass (ICM) of the blastocyst. The three germinal layers (ectoderm, mesoderm, endoderm) are specified through the process of gastrulation. The ectoderm gives rise to the skin and nervous system; the mesoderm gives rise to muscle, bone, blood, and fat; the endoderm gives rise to the respiratory, gastrointestinal, and urogenital tracts. The ectoderm consists of epidermal ectoderm forming the skin, and neuroectoderm, which forms the neural plate and undergoes neurulation to form the neural tube and neural crest cells. The neural tube gives rise to the central nervous system via neural stem cells (NSC), which give rise to neural and glial progenitors, and subsequently neurons, astrocytes, and oligodendrocytes. The neural crest cells migrate and contribute to tissues throughout the body, including the peripheral nervous system (e.g., Schwann cells), ectomesenchymal tissues of the head (e.g., dental pulp stem cells), and the pigmented cells of the skin known as melanocytes. Somatic cells, such as fibroblasts, can be reprogrammed to induced pluripotent stem cells (PSC) through the ectopic expression of four specific transcription factors. These cells can then be differentiated similar to ESC. Somatic cells can also be directly reprogrammed into neurons and NSC. Transdifferentiation is direct reprogramming of cells within the same germinal lineage, such as dental pulp stem cells into neural cells.

Stem cells are unique cells that have the ability to differentiate into a variety of cell types while maintaining the pool of unspecialised stem cells through their self-renewal capacity. They can be classified based on their differentiation potential. Totipotent stem cells can give rise to all the cells needed for embryonic development including the extra-embryonic tissues, while pluripotent stem cells are able to differentiate into cells of all three germinal lineages (ectoderm, mesoderm, endoderm) and hence any cell of the body (Figure 1). Multipotent stem cells are more restricted as they can differentiate into the cells of the tissue they reside in, whereas unipotent stem cells maintain the population of one cell type within their tissue. There are two broad types of stem cells; pluripotent embryonic stem cells and multipotent post-natal adult or somatic stem cells.

Embryonic stem cells (ESC) are derived from the early embryo, more specifically the inner cell mass of the blastocyst (Figure 1). They were first isolated from the mouse blastocyst in the 1980s (8, 9), then from human embryos in 1998 (10). They can be propagated indefinitely and are cultured as embryoid bodies or as a monolayer using feeder layers. Due to their pluripotent nature, ESC give rise to teratomas *in vivo*, which are tumours derived from multiple germ layers and consist of a mixture of differentiated cells (11). By applying specific molecules, ESC can be induced along the neural lineage, making them safer for transplantation (11, 12). However, ESC-derived cells are subject to ethical controversy as culturing ESC involves the destruction of embryos.

Adult stem cells are found in tissues throughout the developed body where they function to maintain homeostasis and repair by giving rise to tissue-specific specialised cells. Examples of multipotent adult stem cells are bone marrow-derived mesenchymal stem or stromal cells (BMSC) and haematopoietic stem cells. Adult stem cells are not subjected to the same ethical considerations as ESC, nor do they possess the same tumorigenic potential. They also allow for autologous cell therapy, to prevent immunocompatibility issues.

It was believed that lineage specification and terminal differentiation into specialised cells was an irreversible process. However, this classic notion that cellular identity is stable has been challenged in the past few decades by evidence of nuclear transfer, dedifferentiation, epigenetic modifications, and ectopic gene expression, suggesting that cells are plastic (13). We now know that somatic cells, such as fibroblasts, can be genetically reprogrammed to stem cells that resemble ESC, termed induced pluripotent stem cells (induced PSC) (14, 15) (Figure 1). Breakthrough research conducted by Yamanaka and colleagues more than a decade ago, demonstrated the underlying molecular mechanism permitting this plasticity (14, 15). Yamanaka's reprogramming method involves the use of specific transcription factors that activate/deactivate genes and modify signalling pathways. In this case, the transcription factors used consist of the pluripotency-associated factors; octamer-binding transcription factor 4 (OCT4), SRY (sex determining region Y)-box 2 (SOX2), KLF4, and MYC. These induced PSC resemble ESC with respect to their morphology, growth properties, gene/protein expression profiles, differentiation potential, teratoma formation *in vivo*, and potential to generate chimeras when injected into blastocysts to demonstrate germline transmission (14, 15). Induced PSC present a cell population with the same level of pluripotency as ESC but circumvent ethical issues, while also providing a source of cells for autologous cell therapies like adult stem cells. However, induced PSC too have a greater risk of tumorigenesis, thus giving adult stem cells, despite their more limited potential, an advantage in terms of safety and timing to the market. Nevertheless, this breakthrough technology has expanded the stem cell research field and helped enhance the progress of cell therapies by providing a cell source with unlimited proliferation and differentiation potentials, and more importantly, a strategy for manipulating cells across all cell lineages.

Within a few years of discovery, the focus on cellular reprogramming turned to a direct strategy. The concept was to bypass the pluripotent state and reprogram cells directly to the desired cell type, thus providing an accelerated reprogramming pathway with a biologically safer product. This direct reprogramming pathway is sometimes referred to as transdifferentiation. In theory, transdifferentiation occurs within the same germinal lineage (Figure 1) (13), thereby requiring fewer epigenetic modifications and decreasing the risk of accumulating mutations and leading to tumorigenesis. Nevertheless, some studies have claimed that inter-lineage transdifferentiation is possible via various transient intermediates (16). Direct strategies therefore increase safety and efficiency, and result in terminally differentiated cells or multipotent stem/progenitor cells with limited proliferation and differentiation capacity (Figure 1). This strategy has been applied to different cell sources to induce cells along the neural lineage (6, 13).

## Neural Stem Cells

The CNS has an inherent, albeit limited capacity for repair. Neural stem cells, capable of self-renewal and differentiation into neurons, astrocytes, and oligodendrocytes, reside in specific regions called neurogenic niches. The formation of the CNS is a series of complex developmental processes initiated early in embryonic development, following the specification of the three germ layers, the ectoderm, mesoderm and endoderm. The neuroectoderm is induced by the underlying mesoderm, through the inhibition of signals that specify epidermal ectoderm. Neurulation is then initiated when the neural plate folds in to form the neural tube, which is composed of neuroepithelial cells (Figure 1). These cells give rise to bipolar NSC called radial glia, which initially span the neural tube, multiply and act as scaffolds for migrating cells. They give rise to cells that respond to gradients of morphogens and mitogens, resulting in the patterning of the neural tube and ultimately giving rise to all the neurons and glia of the CNS (17).

The adult mammalian brain has two main neurogenic niches that contain NSC; the sub-ventricular zone of the lateral ventricles and the sub-granular layer of the dentate gyrus in the hippocampus (17). Neural stem cells give rise to intermediate glial and neural progenitors. Subsequently neuroblasts arise from neural progenitors, and migrate and differentiate into various neuronal sub-types that may integrate within the existing network. Under normal physiological conditions, neuroblasts in the sub-ventricular zone migrate to the olfactory bulb via the rostral migratory stream after which they migrate radially and differentiate into interneurons (granule cells and periglomerular cells), while those in the sub-granular layer migrate into the inner granule cell layer and differentiate into granule cells (17). Adult neurogenesis has been proposed to be involved in maintenance and repair of the neural network, memory formation, and olfaction (17).

Neural stem cells are mostly quiescent under normal physiological conditions but are induced to proliferate and migrate to the affected site in response to injury and disease, such as stroke (17). It has been shown that stroke induction stimulates NSC proliferation and neuroblasts are recruited to the ischaemic striatum (18, 19). This migratory response is mediated by molecules, such as the chemokine stromal cell-derived factor 1-alpha (SDF1α), secreted by glial, immune and endothelial cells (20–22). However, due to the hostile microenvironment, cell survival is low, thereby limiting the intrinsic repair potential (17).

Neural stem cells were described and isolated from the adult mouse brain in the late 1980s (23) and early 1990s (24), though there was evidence of mitogenic cells in the rodent brain from as early as the 1960s (25). In the presence of growth factors, including basic fibroblast growth factor (bFGF) and epidermal growth factor (EGF), these cells proliferated and formed cell clusters termed neurospheres, which could be passaged following single-cell dissociation to generate second, third and further generation neurospheres, or differentiate into neurons and glial cells (24). They were also capable of surviving, proliferating and differentiating when transplanted into the CNS (26, 27). In the late 1990s, analysis of post-mortem tissue confirmed the presence of NSC in the adult human brain (28, 29) and a few years later, NSC were isolated from the adult human olfactory bulb from patients undergoing invasive neurosurgery (30). They are rarely accessible from the living adult human brain and only through extremely invasive surgery, therefore NSC from rodents have been studied as well as human foetal brain-derived NSC (31, 32).

It is presumed that the optimum brain repair mechanism resides within the brain mediated by the local stem cells. Age is a risk factor for neurological diseases including stroke, but aging also attenuates brain repair potential, thus increasing susceptibility to disease. Neural stem cell proliferation rate, viability, and migration capacity can thus be restricted (17). For this reason, exogenous sources of NSC are being studied, including ESC-derived, foetal brain-derived, or reprogrammed from somatic cells either indirectly via the production of induced PSC or directly bypassing the pluripotent stage (6). Ethical and practical considerations limit the application of NSC from ESC and brain tissue, leaving reprogramming as an appealing alternative to provide a source of NSC for cellular therapy.

To be applicable for regenerative medicine, a cell source needs to be easily accessible, expandable under good manufacturing practices to provide sufficient cell numbers, and able to be differentiated/reprogrammed using an efficient and reproducible protocol with defined media. In this regard, stem cells have been investigated, and promising candidates include neural crest-derived DPSC (7, 33).

## Dental Stem Cells

**Figure 2:**
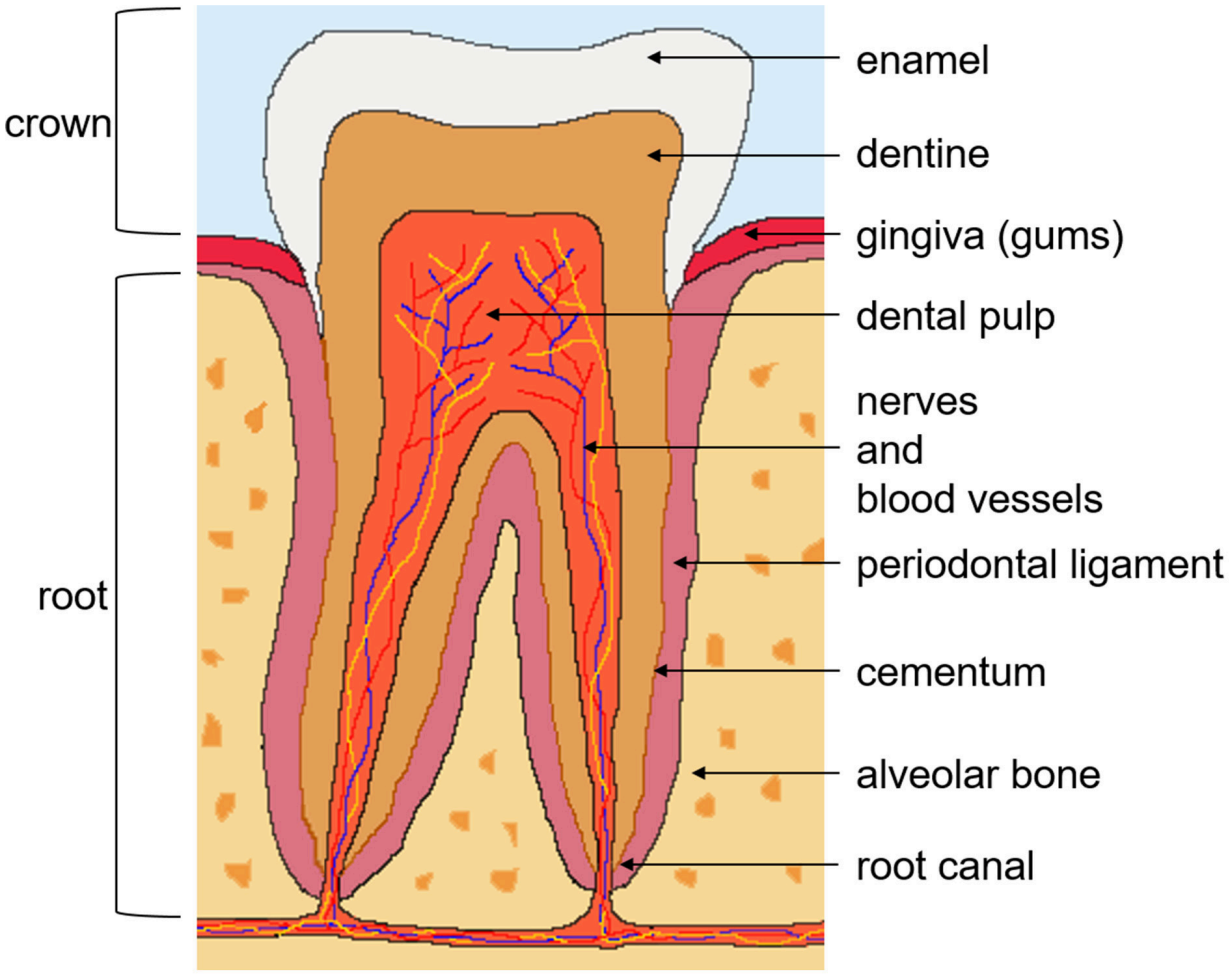
Tooth anatomy and dental pulp stem cells. There are two anatomical parts of the tooth; the crown exposed to the oral cavity and the root embedded in the gum. The crown is composed of enamel on the outside, the underlying dentine, and pulp tissue, which contains nerves, blood vessels, and lymphatics. The root, covered with the bone-like tissue cementum, contains dentine and the root canal. The tooth is supported by the periodontal ligament, connecting it to the alveolar bone. Dental pulp stem cells reside in the perivascular niches of the dental pulp tissue of permanent teeth.

The tooth is an ectomesenchymal tissue that contains, along with the supporting tissues, multiple stem cell niches. Several dental stem cell populations have been identified, which include; DPSC from the dental pulp tissue of permanent teeth (Figure 2) (7), stem cells from the dental pulp tissue of exfoliated deciduous teeth (34), stem cells from apical papilla isolated from the root canal of immature permanent teeth (35), and stem cells from tooth germs (36). Other stem cell populations from the supporting tissues in the oral cavity include; periodontal ligament stem cells (37), alveolar bone-derived MSC (38), gingival MSC (39), and dental follicle stem cells from the surrounding connective tissue of the tooth germ (40).

Dental stem cells follow the criteria that define multipotent mesenchymal stem/stromal cells (MSC), as described by the International Society for Cellular Therapy in 2006 (41). These criteria include; plastic adherence under standard culture conditions; ability to differentiate along the adipogenic, chondrogenic, and osteogenic lineages when cultured in appropriate inductive media; expression of the common MSC-associated markers CD73, CD90, CD105; and lack of expression of haematopoietic markers CD14, CD34, CD45, CD19, and HLA-DR. These dental stem cells are isolated similarly to MSC-like populations from other tissues, such as bone marrow and adipose tissue, and display a spindle-shaped morphology. This review will focus on DPSC, which were the first dental stem cells to be identified.

## Dental Pulp Stem Cells

Human DPSC isolated from third molars, were first described by Gronthos et al. (7). They are a heterogeneous multipotent adult stem cell population that reside in the perivascular niche of the dental pulp (Figure 2) (42). The role of the dental pulp is to maintain tooth homeostasis and repair. The dental pulp cell population consists of dentine-generating odontoblasts on the outer side of the tissue, fibroblasts, immune cells, nerve and glial cells, and stem and progenitor cells in the perivasculature. It was demonstrated early on that DPSC can regenerate tooth structures, as they were capable of forming a dentin-pulp complex when transplanted into immunocompromised mice, which suggested the presence of stem and progenitor cells (7).

Dental pulp stem cells possess high proliferative and clonogenic capacity, and it is for this reason that they have attracted attention with regard to therapeutic applications (7). Limited cell proliferation and tendency to undergo senescence when cultured *ex vivo*, is a challenge that needs to be overcome if cells are to be used in the clinic. In comparison to some cell populations, such as BMSC, DPSC display a higher frequency of colony formation and greater proliferation potential, which is maintained throughout culture (7, 43).

Their multilineage differentiation potential has been demonstrated under *in vitro* inductive conditions and following implantation *in vivo*, along multiple lineages including the odontogenic, osteogenic, chondrogenic, adipogenic, neurogenic, and myogenic lineages (44, 45). This extensive array of lineages is an advantage over other stem cell populations, such as haematopoietic stem cells, which have a more limited differentiation capacity.

There is no known unique marker to identify and isolate DPSC. Extensive immunophenotyping of *ex vivo* expanded DPSC has demonstrated their expression of markers associated with; MSC-like populations; bone and dentine; and perivascular cells, including CD146 (7). Dental pulp stem cells may also express neural lineage markers, including low-affinity nerve growth factor receptor known as p75, the intermediate filament nestin, and glial fibrillary acidic protein, as well as more mature neuronal lineage markers, such as β-III tubulin and neuronal nuclear antigen (45). This immunophenotype reflects their origin and high level of heterogeneity.

Dental pulp stem cells originate from the embryonic neural crest. In vertebrate embryogenesis, during the formation of the neural tube, a transient population of multipotent cells arises at the junction between the neural tube and the epidermal ectoderm, termed the neural crest (Figure 1). These neural crest cells migrate from the dorsal margins of the neural tube, undergoing an epithelial to mesenchymal transition, and give rise to multiple neural and non-neural cell types throughout the body, including all of the neurons and glia of the peripheral nervous system, the ectomesenchymal derivatives of the craniofacial area and melanocytes of the skin (Figure 1). It is the migrating cranial neural crest cells that contribute to the dental pulp tissue, established using genetic lineage tracing (46–48). In addition, a more recent study demonstrated that a significant subpopulation of cells extracted from the dental pulp originates from peripheral nerve-associated glia during tooth development, homeostasis and regeneration (49). Thus, neural crest-derived stem and progenitor cells persist in adult tissues, and some, such as the glial Schwann cells lining peripheral nerves and melanocytes in the skin, are able to dedifferentiate into stem/progenitor-like cells (13).

It is therefore not surprising that DPSC express markers of the neural lineage, which substantiate their neural predisposition. However, they are heterogeneous and this quality can impact the differentiation efficiency (7). Analysis of individual colonies also demonstrated the different proliferation rates, suggesting that the more highly proliferating cells will dominate a multi-colony population, altering the composition (33).

Most studies have focused on using the whole DPSC population extracted from the dental pulp (50, 51). However, it may be beneficial to select subpopulations, using fluorescence- or magnetic-activated cell sorting, based on markers that may be associated with increased neurogenic potential. Using a more homogenous cell population could increase the differentiation efficiency. For example, stromal precursor cell surface marker STRO-1, known as an early marker of multiple MSC-like populations, has been used to purify DPSC (42). However, STRO-1 is downregulated very early during *ex vivo* expansion, creating a problem in obtaining sufficient cell numbers (42). The neural crest marker p75, co-expressed with STRO-1 on BMSC (52), may be suitable as p75^+^ DPSC express higher levels of neural stem cell markers (53). It is however expressed by <10 percent of the overall DPSC population (53). Isolation methods may also make a difference. The common isolation method is to enzymatically release the cells from the dental pulp tissue using collagenase and dispase (7). Alternatively, cells can be allowed to migrate out using the explant method, which may select for specific subpopulations (54).

Dental pulp stem cells have also been shown to express the pluripotency-associated markers OCT4, SOX2 and MYC (55), which is uncommon to MSC. However, unlike ESC and induced PSC that express these markers, transplanted DPSC have not resulted in tumour formation (33). Additionally, both spontaneously and induced immortalised DPSC did not form tumours when transplanted into immunocompromised mice (56). Embryonic stem cells and induced PSC require complete differentiation prior to implantation due to tumorigenic risk.

These properties make DPSC promising candidates for stem cell therapy, in particular for neurological diseases due to their neurogenic predisposition. Most importantly, dental pulp presents an easily-accessible non-invasive source of adult stem cells with high proliferation potential. They are isolated from routinely extracted teeth that would otherwise be discarded as clinical waste, unlike the invasive aspiration of bone marrow required to obtain BMSC.

## Neuronal Differentiation of Human Dental Pulp Stem Cells

Under defined neuronal inductive conditions, human DPSC are able to differentiate into functionally active neuronal cells. They acquire a neuronal morphology, displaying a rounded cell body with cytoplasmic extensions, and show an immunophenotype consisting of neuronal-associated markers (e.g., β-III tubulin, neuronal nuclear antigen) (50, 51). As outlined in Table 1, there have been multiple neuronal differentiation protocols applied to human DPSC, varying in media composition, use of supplements, growth factors, and small molecules, as well as seeding density, growth surfaces, number of stages, and duration. However, few studies have conducted functional analysis of their neuronally differentiated DPSC (Table 1), such as electrophysiological analysis for the presence of voltage-gated ion channels (Na^+^, K^+^, Ca^2+^), which are required for the generation and propagation of action potentials in functionally mature neurons.

**Table 1:** Neuronal induction protocols for human dental pulp stem cells with functional assessment.

**Cells**	**Growth conditions**					**Functional assessment**	**References**
	**Basal media**	**Use of spheres**	**Coating**	**Growth factors**	**Duration**		
CD34^+^/cKit^+^/ STRO-1^+^ DPSC	DMEM/F12	Yes	Poly-L-lysine	bFGF EGF BDNF NGF	>3 weeks	Patch-clamp analysis of Na^+^ and K^+^ currents.	Pisciotta et al. (67)
DPSC	DMEM/F12	Yes	Poly-L-ornithine, laminin	bFGF EGF BDNF GDNF NT-3	19–23 days	Fluorescent detection of Ca^2+^ influx following stimulation.	Gonmanee et al. (63)
DPSC	Neuro-basal	No		bFGF EGF BDNF	12 days	Fluorescent detection of Ca^2+^ influx following stimulation.	Singh et al. (62)
DPSC	DMEM/F12	No	Type I and IV collagen, laminin, fibronectin	bFGF EGF BDNF GDNF IGF-I	>3 weeks	Transplantation into neonatal rat brain and spinal cord injury rat model.	Jung et al. (64)
DPSC	Neuro-basal A	No	Geltrex	bFGF EGF	3 weeks	Patch-clamp analysis of Na^+^ and K^+^ currents.	Ullah et al. (58)
DPSC	DMEM/F12, Neuro-basal	Yes	Poly-L-ornithine, laminin	bFGF EGF NT-3	5 weeks	Patch-clamp analysis of Na^+^ and K^+^ currents, and action potential production.	Gervois et al. (65)
DPSC	Neuro-basal	No		bFGF FGF8 BDNF	9 days	Fluorescence detection of Ca^2+^ influx, and measurement of dopamine release with/without stimulation.	Kanafi et al. (61)
DPSC	Neuro-basal	Yes	Type IV collagen	bFGF EGF	2 weeks	Fluorescent detection of Ca^2+^ influx following stimulation.	Osathanon et al. (66)
DPSC	DMEM/F12, Neuro-basal A	No	Poly-L-lysine	bFGF NGF NT-3	9–13 days	Patch-clamp analysis of Na^+^ and K^+^ currents, transplantation into the cerebrospinal fluid of neonatal rats, transplantation into rats with lesions to the forelimb motor cortex.	Kiraly et al. (51, 59)
DPSC	Neuro-basal A	No	Poly-ornithine, laminin	bFGF EGF	3 weeks	Patch-clamp analysis of Na^+^ currents.	Arthur et al. (50)

*BDNF, brain-derived neurotrophic factor; bFGF, basic fibroblast growth factor; DMEM, Dulbecco's Modified Eagle's Medium; DPSC, dental pulp stem cell(s); EGF, epidermal growth factor; F12, Ham's F12 nutrient mixture; FGF8, fibroblast growth factor 8; GDNF, glial cell-derived neurotrophic factor; IGF-I, insulin-like growth factor I; NGF, nerve growth factor; NT-3, neurotrophin-3*.

One of the earlier methods for neuronal induction of human DPSC, and the first to examine their functionality, resulted in neurons that displayed voltage-gated sodium channels, important for initiation of action potentials (50). A subpopulation of DPSC has been shown to display sodium currents in their normal state (57). These cells may have a role in sensory transduction within teeth. If this subpopulation differentiates into neuronal-like cells, enhanced electrophysiological properties would be expected. It is however uncertain, which subpopulations neuronally differentiate, and whether they initially display any voltage-dependent ion currents. A modified version of this neuronal differentiation protocol resulted in cells expressing both sodium and potassium currents (58).

Subsequently, a shorter, 2-week neuronal differentiation protocol for DPSC was developed, resulting in neurons displaying both voltage-dependent sodium and potassium currents and were sensitive to respective inhibitors (51). This method had a pre-induction step exposing the cells to the demethylating agent 5-azacytidine, thus modifying the epigenome to increase the ability of the cells to go down the specified lineage. These neuronally differentiated human DPSC localised to the neurogenic niches in the rodent brain and were detected in the lesioned cortex following injury (59). This demonstrated that the DPSC-derived neuronal cells survived and responded to the environmental signals, similar to results obtained in an avian embryo model using undifferentiated human DPSC (60).

Other groups have been interested in directing DPSC into specific neuronal types, including dopaminergic and spiral ganglion neurons (61–63). However, the investigations are few at the current time and more research is needed. Furthermore, xeno- and serum-free conditions from initial culture to neuronal differentiation have also been investigated (64). Removing animal-derived products and creating defined media is a critical step towards establishing a safe and consistent cell source for therapeutic purposes.

More recently, studies have begun to use neurosphere generation for neural induction of human DPSC (65). Neurospheres are a commonly used culture system for NSC, where cells are grown in conditions lacking adherent substrates and in the presence of growth factors, most commonly bFGF and EGF. They are heterogeneous, containing cells at different stages of differentiation and maturation, thus identifying the presence neural stem cells. Recent studies have incorporated this culture system in the neuronal differentiation of DPSC (65, 66). The cells were differentiated to functional neurons *in vitro*, through a two-step protocol, firstly by stimulating neurosphere formation, then going through a neuronal maturation stage. Patch-clamp analysis and calcium imaging were used to confirm the presence of functional properties. One research group observed that the cells not only expressed functional voltage-gated sodium and potassium channels but a subset of cells also generated single action potentials (65). Repeated action potentials were not observed nor were spontaneous action potentials, which would indicate fully functional neurons. The initial neurosphere culture step was further analysed by Pisciotta et al. (67), who demonstrated the prolonged expansion of the DPSC-derived spheres throughout which they maintained their neural crest properties.

These *in vitro* differentiation studies demonstrated that DPSC respond to local environmental cues, which is also true for the *in vivo* setting. When injected into an embryonic model at a time of active neurogenesis, DPSC followed the migratory pathway of endogenous cranial neural crest cells, reiterating their origin, and underwent neuronal differentiation (60).

## Human Dental Pulp Stem Cells in Stroke Models

Despite the extensive *in vitro* research examining the neurogenic properties of DPSC, there have only been a hand-full of studies that have examined the efficacy of human DPSC-based therapies *in vivo* to treat stroke. Table 2 summarises the use of human DPSC in animal stroke models, based on rodent models of focal cerebral ischaemia via the transient occlusion of the middle cerebral artery.

**Table 2:** Human dental pulp stem cells in stroke models.

**Cells**	**Experimental model**	**Delivery method**	**Cell number**	**Timing**	**Outcome**	**References**
DPSC	Rat Transient Middle Cerebral Artery occlusion (MCAo)	IV	1 × 10^6^	Cells injected at 0 or 3 h post-stroke, analysis performed up to 14 days post-stroke.	Cells reversed motor deficits and reduced infarct volume by inhibiting microglial activation, pro-inflammatory cytokine production, and neuronal degeneration.	Nito et al. (71)
DPSC over-expressing hepatocyte growth factor		IV	1 × 10^6^	Cells injected at 0 h post-stroke, analysis performed up to 14 days post-stroke.	Cells improved motor function and decreased infarct size via immunomodulation, enhanced angiogenesis, suppression of neuronal degeneration, and maintenance of blood-brain barrier integrity.	Sowa et al. (76)
CD34^+^/c-Kit^+^/ STRO-1^+^ DPSC		IV	4 × 10^6^	Cells injected 24 h post-stroke, analysis performed up to 28 days.	Cells stimulated functional recovery and decreased infarct volume via autocrine/paracrine mechanisms.	Song et al. (75)
DPSC		IC	6 × 10^5^	Cells injected 24 hours post-stroke, analysis performed up to 28 days.	DPSC enhanced post-stroke forelimb sensorimotor recovery via DPSC-dependent Paracrine effects.	Leong et al. (69)
Dental pulp-derived nuclear receptor related 1 protein^+^ stem cells		IC	1–2 × 10^5^	Analysis performed up to 28 days.	Transplanted cells survived and promoted functional recovery, due to hypo-immunogenic properties and immunomodulation ability.	Yang et al. (68)

*DPSC, dental pulp stem cell(s); IC, intracerebral; IV, intravenous*.

The first study to investigate DPSC in an animal model of stroke used a mechanical extraction method to obtain cells from human wisdom teeth (68). These cells expressed the nuclear receptor related 1 protein, which is essential for the dopaminergic system of the brain. Although the study involved a small sample size, it provided preliminary data for the therapeutic potential of dental pulp cells for stroke. Functional recovery was exhibited and cells were detected in the penumbra, however potential mechanisms were not investigated.

A subsequent study provided the first preclinical support for use of human DPSC in acute ischaemic stroke (69). Dental pulp stem cells were intracerebrally transplanted into the rat brain 24 h post-stroke, at two injection sites; in the cortex and in the striatum. At 4 weeks, significant neurobehavioural improvement was observed, however only 2.3 percent of engrafted cells were detected. These results suggest that the improvement was not mediated by a cell replacement mechanism rather through a DPSC-dependent paracrine effect, involving the many growth factors secreted by DPSC, such as nerve growth factor, brain-derived neurotrophic factor, glial-derived neurotrophic factor, and ciliary neurotrophic factor (70, 71). The low survival rate observed in this preclinical model may indicate a specific subpopulation of DPSC have greater neurogenic potential and ability to survive and differentiate, with the p75^+^ subpopulation being a candidate. The DPSC displayed targeted migration towards the infarct, and differentiated into astrocytes within the vicinity of the infarct and neurons further from the infarct site. The targeted migration of the transplanted cells towards the ischaemic border zone is likely mediated by the SDF1 gradient produced by endogenous cells following stroke (20). This chemokine is upregulated for at least a month post-stroke (72). Furthermore, it was previously shown in an avian embryo model that DPSC induce endogenous axon guidance of host trigeminal ganglion axons via the production of SDF1 (60). In this preclinical study, a reduction in post-stroke corpus callosum atrophy was also observed, again indicating to the ability of DPSC to influence host axonal remodelling. These results demonstrate the role of SDF1 signalling in DPSC migration and neuroplasticity properties.

Of note, both of these studies used an intracerebral route of administration, which is a highly invasive procedure. Subsequent studies used an intravascular route, which may be clinically safer in the acute stroke setting (73). It has been shown that stem cells administered through this route can be detected in the brain but involves the transmigration of the BBB. This could be a result of the disrupted BBB post-stroke, however cellular trafficking occurs even after the period of increased BBB permeability (74), implicating other mechanisms at play.

In these DPSC studies, administration via the tail vein resulted in cells transmigrating the BBB and migrating towards the infarct (71, 75, 76), with a lack of detection in systemic organs (75). Winderlich et al. previously demonstrated that human DPSC stimulate BBB permeability via the production of the soluble factor vascular endothelial growth factor-alpha (VEGFα) (73), which is known to be produced following stroke and is secreted by stem cells, including DPSC (77). DPSC mediate their angiogenic effect via VEGFα (75, 76). In addition to its role in angiogenesis, VEGFα is involved in vascular permeability, and has been shown to decrease tight junction proteins thus increasing permeability (78). Interestingly, Sowa et al. showed that the integrity of the BBB was maintained via the DPSC attenuation of the decrease in tight junction proteins that results from stroke (76). This was observed with unmodified DPSC, but also with DPSC overexpressing the pleiotropic cytokine hepatocyte growth factor, which showed an even greater effect. This factor may have a role in inhibiting BBB disruption (79). Hence, DPSC not only produce factors like VEGFα that allow them to transmigrate the BBB but they also have the counteracting potential to maintain BBB integrity. There are likely other factors involved in regulating BBB permeability and with more research, the complex mechanisms used by stem cells can be uncovered.

Dental pulp stem cells have been shown to modulate the immune response in several ways. They inhibit microglial activation, as evidenced by the decrease in cells expressing ionised calcium-binding adaptor molecule 1, a marker specifically expressed in the brain by activated microglial cells (71, 76). Analysis of DPSC-conditioned medium has shown their expression of various cytokines, including interleukin 10, which decreases pro-inflammatory cytokine production (71). Additionally, decreased levels of the pro-inflammatory cytokines tumour necrosis factor-alpha and interleukin 1-beta are observed in brain tissue and serum from DPSC-treated stroke animals (71, 76).

The BBB has a role in maintaining the immune privileged status of the CNS. It appears that DPSC are not entirely rejected, as these studies have not employed the use of immunosuppressive drugs (71, 75, 76). This could be mediated by the DPSC expression of the Fas ligand, which induces the apoptosis of T cells, and is also one of the mechanisms used to maintain immune privilege of certain tissues. Fas ligand is known to be expressed by stem cells including DPCS, even with prolonged culture and particularly using the neurosphere culture system (67). Thus, DPSC have the potential to evade the immune system, transmigrate the BBB, migrate towards the infarct and modulate the inflammatory response. The ability of DPSC to exert these therapeutic effects in the acute phase when inflammation occurs, demonstrates their high level of potency (71).

Dental pulp stem cells are also able to inhibit reactive astrogliosis (75), which leads to glial scar formation as a protective mechanism but impedes axonal regrowth. Attenuation of neuronal degeneration by DPSC is also evidenced by decreased numbers of degenerating neurons (71, 76). This neuroprotective effect is likely mediated by the array of neurotrophic factors produced by DPSC, such as nerve growth factor, brain-derived neurotrophic factor, glial-derived neurotrophic factor (70). Additionally, DPSC express the cytokine interleukin 3 (IL-3) (71), which promotes proliferation and differentiation of stem cells, suggesting that DPSC may have capacity to stimulate endogenous cells, though this mechanism has not been investigated in these studies.

**Figure 3:**
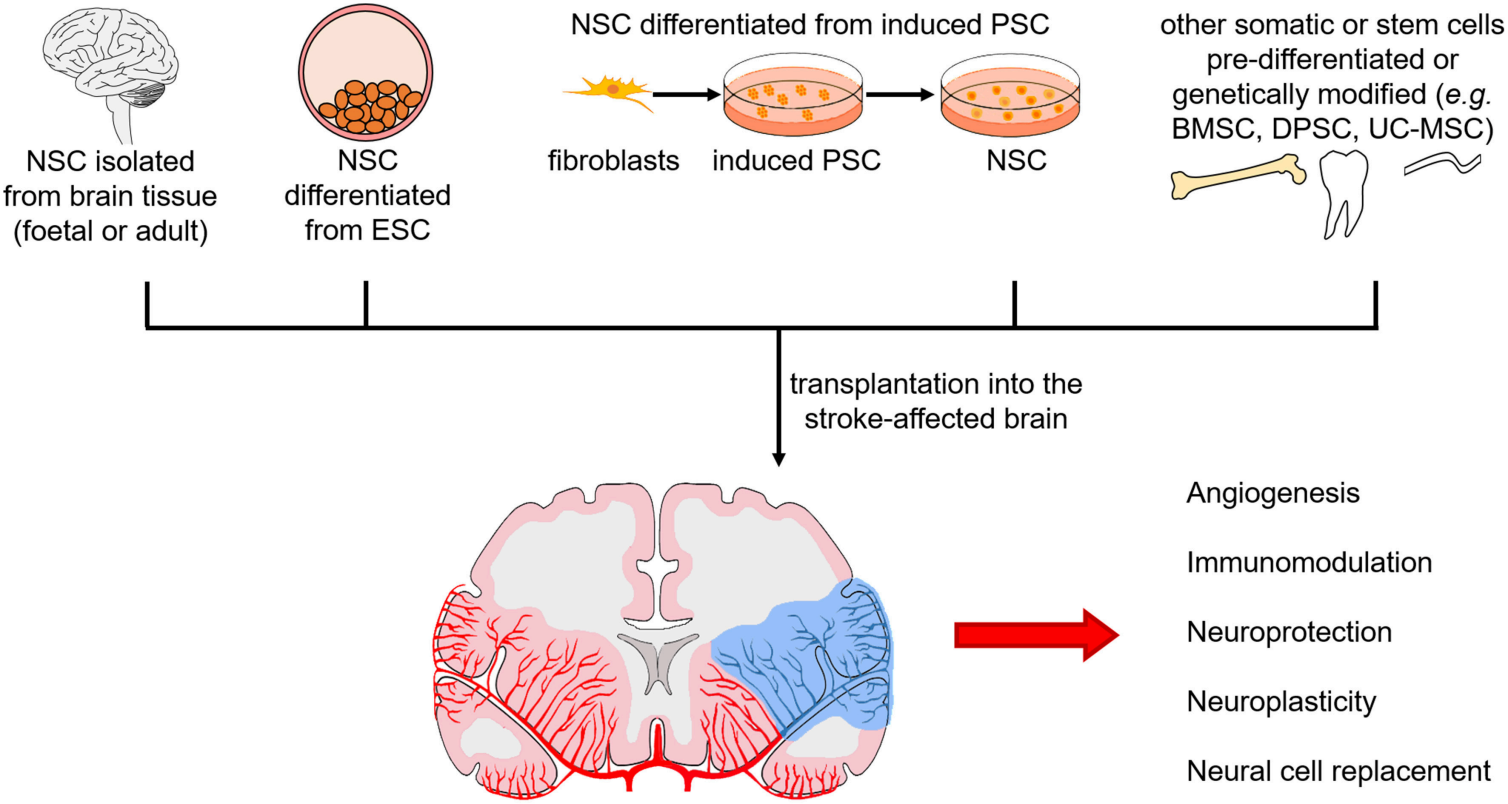
Sources of neural stem cells for transplantation in the stroke-affected brain and mechanisms of action. Neural stem cells (NSC) from foetal and adult brain tissue, differentiated from pluripotent stem cells [embryonic stem cells (ESC) or induced pluripotent stem cells (PSC)], and differentiated or genetically modified from other somatic cells and stem cells [e.g., bone marrow stem cells (BMSC), dental pulp stem cells (DPSC), umbilical cord-derived mesenchymal stem cells (UC-MSC)], are being investigated. Mechanisms of action in the stroke-affected brain include stimulating angiogenesis, modulating the immune response, providing neuroprotection, inducing neuroplasticity, and replacing neural cells.

These *in vivo* studies observed functional recovery and decreased infarct volumes (Table 2). Evidence from all preclinical studies suggest that the therapeutic effects of DPSC are most likely mediated through paracrine mechanisms and less so of a neural cell replacement mechanism. Dental pulp stem cells secrete growth factors, neurotrophins and cytokines, which promote angiogenesis, modulate the immune response, provide neuroprotection, and enhance neuroplasticity (Figure 3).

Even though cell survival and differentiation to astrocytes and neurons is observed, neural tissue replacement is unlikely to play a big part. Cell survival may be increased through the addition of neurotrophic factors, such as brain-derived neurotrophic factor (80). Alternatively, the culture method may be important, with a better outcome observed with DPSC-derived neurospheres, possibly due to greater amounts of neural progenitor cells (81). As previously mentioned, certain DPSC subpopulations may have greater neurogenic potential and survival. The perivascular cell marker CD146 is used to purify MSC-like populations from different tissues. However, it may actually select cells that are more committed to non-neural lineages, as CD146^+^ cells demonstrate greater regeneration of the dentin-pulp complex than CD146^−^ cells (82). The CD146^−^ subpopulation may instead contain cells that are of neural crest origin, and may explain why it displayed positive outcomes in an ischaemic model (83). Another investigated subpopulation is CD34^+^ DPSC, which despite their slower proliferation and loss of “stemness,” have greater commitment to the neural lineage and express the neural markers p75 and nestin, compared to CD34^−^ DPSC (75, 84). Although CD34 is believed to be a distinct marker for cells of haematopoietic origin and endothelial cells, this subpopulation improved functional recovery via the aforementioned mechanisms but also exhibited superior neuroprotective properties in comparison to BMSC (75).

There is strong experimental evidence that stem cell therapy can improve neurological function through a variety of cellular and molecular mechanisms, with growing evidence to support DPSC as an attractive source. These cells are appealing for use in autologous and allogeneic therapies of stroke. Stroke is more widely regarded as an aging disease, and improved healthcare suggests that a greater proportion of the older population retain their teeth. Nevertheless, to reach all stroke patients irrespective of having their own teeth, an allogeneic strategy may be required, and the immunosuppressive properties of DPSC make them attractive for this type of clinical application (85).

While promising, the efficacy of DPSC to deliver therapeutic outcomes based on their capacity to differentiate into functional neural cell populations remains the subject of intense research. The limitation in using undifferentiated and unselected DPSC is that they may differentiate along multiple lineages or only a small fraction would differentiate to neural cells, thereby decreasing the clinical efficacy. A neural cell population would be more suited to the environment and have greater potential to replace lost and damaged neural tissue. For example, neuronally pre-differentiated human DPSC responded well to the normal and injured *in vivo* environment (59). However, since neuronal cells lack the ability to self-renew, the number of cells that can actually mediate positive outcomes will be fewer than initially transplanted due to cell loss. Furthermore, in a disease like stroke where the ischaemic milieu affects multiple neural cell populations, administering neuronal cells would be targeting the replacement of only one affected population. Neural stem or progenitor cell populations would be a better alternative, allowing expansion to provide sufficient cell numbers and differentiation into various cells based on the endogenous environment.

## Reprogramming Dental Pulp Stem Cells

Finding an alternative source of NSC has been a major research focus for some years now, with much of the research investigating cellular reprogramming strategies. In this regard, DPSC might be a promising cell source. Due to their neural crest origin, they may require less manipulation or display greater efficiency than other cell types. As previously mentioned, cells can be directed along the neural lineage either indirectly, using induced PSC production, or directly to the desired cell type.

Dental pulp stem cells have successfully been reprogrammed to induced PSC (86, 87), even in the absence of the oncogenic MYC, and subsequently directed along the neural lineage (88). Dental pulp can thus provide an alternative cell source for establishing induced PSC banks for regenerative medicine. There has been little investigation in the area of converting DPSC into NSC, other than by the use of neurosphere culture methods, usually as a transient step in the pathway to neuronal differentiation (65). While there has been some evidence of the neurosphere culture system maintaining prolonged culture of DPSC-derived spheres (67), these spheres are not clonal nor consisting of only stem cells. Since DPSC are of neural crest origin, it may be more efficient to use a direct reprogramming strategy to convert them to NSC, in comparison to cells of a different germinal lineage. Transdifferentiation should be possible between these cell types, which share a common tissue of origin. Similar methods that have been applied to other somatic cell types to induce NSC can be applied to DPSC (89–92).

## Direct Reprogramming of Cells Along the Neural Lineage

Direct reprogramming along the neural lineage was first investigated by Wernig et al. who demonstrated the production of induced neurons from mouse fibroblasts using the neural-specific transcription factors, ASCL1, BRN2 and MYT1L (93), and from human fibroblasts with the addition of the NEUROD1 transcription factor (94). Since fibroblasts are from a heterogeneous mesenchymal cell population potentially containing cells of neural crest origin, they further applied this transcription factor combination to a homogeneous non-neural-derived cell population (95). This demonstrated that reprogramming is not restricted to cells of the same germinal lineage. Thereafter, they established that the pro-neural factor ASCL1 alone was capable of reprogramming fibroblasts to neuronal cells (96); thus this gene may be viewed as a pioneer transcription factor (97). Reprogramming is associated with epigenetic modifications of the DNA. Pioneer transcription factors are first to occupy chromatin sites, leading to remodelling of the chromatin configuration at lineage-specific genes and subsequent recruitment of other transcription factors, demonstrating the hierarchical mechanisms in lineage reprogramming. The achievement of generating induced neurons is of clinical importance with regard to addressing the safety issues associated with induced PSC. However, the post-mitotic nature of neurons may not be ideal for all applications.

Subsequent studies applied a direct reprogramming strategy to obtain expandable neural stem and progenitor cells, using pluripotency-associated and/or neural-specific transcription factors. The first study to derive induced neural stem or progenitor cells, applied transient expression of the Yamanaka factors (OCT4, SOX2, KLF4, MYC) in fibroblasts. However, these induced cells occurred at a low frequency of < 1 percent, were expandable *ex vivo* for only three to five passages, giving rise to neurons and astrocytes but not oligodendrocytes (16). A similar study used the same factors but limited OCT4 expression to the first 5 days of reprogramming, resulting in tripotent NSC that could be stably expanded for more than 50 passages and exhibited a transcriptional profile similar to brain-derived NSC (92). These studies claimed to transdifferentiate the fibroblasts to the neural lineage using the same factors for induced pluripotency but under neural conditions, thus bypassing the pluripotent state. However, genetic lineage tracing studies demonstrated that these methods actually involved cells passing through a transient pluripotent state, before differentiating along the lineage specified by their environment (98, 99). This suggests that the pluripotent state is a requisite when reprogramming cells between different germinal lineages, although transcription factor combinations may also influence this.

Different research groups instead used neural-specific transcription factor combinations, including SOX2, which has a role in embryonic development and neural induction (89, 100). One combination included BRN2, a transcription factor in the same family as OCT4, which was necessary for tripotency, while SOX2 was essential for development of mature neurons (89). In another study, the Yamanaka factors were used but OCT4 was replaced with BRN4, again in the same family (90). These fibroblast-derived NSC survived in the mouse brain for up to 6 months and differentiated along the three neural lineages (101). More recently, the same group compared the transcriptome of these induced NSC with forebrain-derived NSC and ESC-derived NSC (102). They showed that all three groups of NSC were distinct from one another, but that induced NSC and forebrain-derived NSC had a greater similarity with each other than with ESC-derived NSC. Moreover, this transcription factor combination resulted in NSC that possessed mostly a caudal identity, while forebrain-derived NSC had a rostral identity and ESC-derived NSC had a less defined regional identity indicating their more immature state. This suggests that different transcription factor combinations could yield NSC suitable for different areas of the brain.

From these studies, it appears that members of the POU domain family of transcription factors, like OCT4, have a critical role in lineage conversion (103). This factor is a fundamental transcriptional regulator of pluripotency. The OCT4 and SOX2 transcription factors heterodimerise; SOX2 binds weakly to DNA while OCT4 contains the POU DNA-binding domain. Together, they govern the activation of genes for pluripotency and concurrently the suppression of genes for differentiation. It has been proposed that OCT4 acts as a pioneer transcription factor to induce plasticity of cells, with subsequent factors and environmental conditions directing the lineage differentiation (103, 104). Indeed OCT4 alone has been used to reprogram fibroblasts (105) and blood cells (106, 107) to NSC, which under neural conditions do not display hallmarks of induced pluripotency (104).

Other groups have demonstrated the production of induced neural cells from fibroblasts using only SOX2 (91), and from blood cells using SOX2 and MYC (108, 109). Like OCT4, SOX2 is a key regulator of pluripotency but is further involved in maintenance of multiple stem cell populations, including NSC, and may be crucial for reprogramming (110). The role of the proto-oncogene MYC in the reprogramming process may involve chromatin remodelling. This factor aids the reprogramming process since removal of MYC lowers the efficiency of induced PSC generation (14, 15).

Cellular reprogramming is a rather inefficient process, requiring ongoing investigations to better understand the process and methods to optimise it. Low efficiency may reflect the stoichiometry of the transcription factors or the need for integration of the transgenes in specific loci (14, 15). Furthermore, the process is stochastic; reprogramming occurs randomly so cells in the population can be at different stages of differentiation and maturity, while incomplete reprogramming may also occur, whereby cells retain epigenetic memory of the donor cells (111). The heterogeneity of the initial cell populations and the molecular state of the cells likely contribute to this process.

Rather than inducing the ectopic expression of transcription factors, microRNAs (112) and small molecules (113, 114) have been applied, which in effect target the enzymes for epigenetic modifications and/or transcription factors and other elements of their signalling pathways to result in similar cellular outcomes as transcription factor-mediated reprogramming. For example, the microRNA miR-124 has a role in regulating the epigenome as well as multiple neuronal differentiation genes (6, 112). Chemical cocktails for reprogramming usually include epigenetic modulators that improve efficiency, such as the histone deacetylase inhibitor valproic acid, and DNA and histone methyltransferase inhibitors (113, 114). Other modulators are used to activate or deactivate specific signalling pathways, such as the Wnt, MAPK/ERK, TGFβ, and hedgehog signalling pathways, to activate appropriate neural genes, promote self-renewal, enhance mesenchymal to epithelial transition, improve viability, decrease cell senescence, and increase efficiency (6, 113, 114). This suggests that multiple signalling pathways have important roles in the reprogramming process and a variety of small molecules can be used to achieve this.

With regard to DPSC, little research exists on transcription factor-mediated reprogramming. Induced overexpression of OCT4A, the OCT4 isoform thought to be the key regulator of pluripotency, enhanced expression of other pluripotency regulators and multilineage differentiation in human DPSC (115). However, the effect on differentiation along the neural lineage was not investigated. Overexpression of SOX2 in DPSC was also investigated, focusing on proliferation, migration, and adhesion, but the effect on multilineage differentiation is unknown (116).

## Neural Stem Cell Therapy for Ischaemic Stroke: Preclinical Studies

Neural stem cells are a promising cell source for establishment of functional neuronal networks following disruption by neurological injury or disease. They would be an appropriate stem cell source for stroke treatment, where the ischaemic environment affects multiple cell types, with additional heterogeneity seen among patients. Preclinical studies using NSC in rodent models of ischaemic stroke have utilised human and rodent NSC derived from ESC, brain tissue, induced PSC and more recently via somatic cell transdifferentiation (Figure 3) (117–120). They have been shown to survive, migrate towards the lesion, differentiate into neurons and astrocytes, decrease infarct volume, integrate, induce neuroplasticity, and stimulate functional improvements via paracrine effects (117, 118, 121–125). Transplanted NSC also induce endogenous NSC to proliferate (119, 120, 126, 127).

Even though beneficial effects have been observed, similar to other stem cells, research is still focused on how to better improve survival of the engrafted cells. Neural stem cells are expected to be better suited for the neural environment, and as such, survival of transplanted human foetal NSC has been shown to be >30 percent at 1 month after transplantation (121, 123), in comparison to the survival of human DPSC at only 2.3 percent (69). Cell survival decreased as time of transplantation post-stroke increased, i.e., hours vs. weeks, however it was still more than 10 times greater than that of DPSC (128), demonstrating their ability to survive in the harsh acute stage. Interestingly, a study that examined the difference of foetal and adult mouse NSC showed that even though both showed therapeutic effects, foetal NSC had a higher survival rate (129), indicating that the adult NSC were mediating their effects through mechanisms other than cell replacement similar to observations with other adult stem cells, such as DPSC. This suggests that there is a difference in foetal and adult stem cells that governs their mechanisms of action.

Multiple studies have shown that survival of transplanted NSC is enhanced when modified to express neurotrophic factors, including nerve growth factor (130), VEGF (131), neurotrophin-3 (132, 133), glial-derived neurotrophic factor (134), brain-derived neurotrophic factor (135, 136), and bFGF (137), or when pre-treated prior to transplantation (138).

Ischaemia produces a harsh environment containing reactive oxygen species, which damage macromolecules in the cells. Some studies have demonstrated that hypoxic preconditioning of cells increases viability (139). The hypoxia-inducible factor 1-alpha (HIF1α) transcription factor is expressed under hypoxic conditions, and one of its downstream targets is VEGF, leading to increased angiogenesis. Alternatively, NSC were modified to express HIF1α to aid their survival (140). In another study, NSC that were modified to overexpress an anti-oxidant enzyme to eliminate reactive oxygen species, showed greater survival following transplantation (141). Thus, NSC may be better primed using mechanisms that activate pathways addressing survival under hypoxic conditions.

Interestingly, in addition to the role in angiogenesis, VEGF has been shown to be involved in transdifferentiation of astrocytes into mature neurons (142). This suggests that an additional beneficial effect of reactive astrogliosis, which has a role in neuroprotection following stroke, is neuronal cell replacement. Furthermore, studies often see differentiation of transplanted stem cells into neurons and astrocytes. Timing of transplantation post-stroke has also been shown to affect differentiation, with earlier time point leading to astrocyte differentiation while later time point leads to neuronal differentiation (143). Thus, astrocytes appear to have a crucial role in the acute stage. Overall, NSC exhibit therapeutic effects but there are multiple ways they can be modified, as well as transplantation parameters, which can alter their survivability and differentiation outcome.

## Neural Stem Cell Therapy for Ischaemic Stroke: Clinical Trials

There have been few clinical trials for ischaemic stroke using human neural stem or progenitor cells, and no trials utilising DPSC to date.

The first clinical trial of cell therapy for chronic stroke in patients with fixed motor deficits was conducted using the NTERA2 human teratocarcinoma cell line as a source of neurons. When treated with retinoic acid, these cells that resemble neuroepithelial cells differentiate into post-mitotic neurons that are indistinguishable from ESC-derived neurons. Following the phase I trial consisting of 12 patients and demonstrating the feasibility, a phase II trial was carried out with 18 patients (50 percent ischaemic stroke) who participated in a subsequent stroke rehabilitation program. The safety and feasibility of using these neurons was demonstrated, and although functional improvements were observed, they did not appear to have an overall significant effect on motor function (144, 145).

Several clinical trials have examined multiple cell combination therapy, including neural stem/progenitor cells.

Rabinovich et al. used cells from human foetal nervous and hemopoietic tissues (146). The clinical trial consisted of 10 patients, 5 male and 5 female, aged between 35 to 56 years, with haemorrhagic or ischaemic stroke (70 percent ischaemic stroke) and stable disability 4 to 24 months post-stroke. All patients had received rehabilitation therapy but showed no improvement. Cells were administered in the subarachnoidal space through spinal puncture. There were no serious complications and positive outcomes were observed in all patients, with significant improvements at 6 months compared to the control group. Positive shifts in neurological status became apparent at 2–3 weeks and progressed in the subsequent 30–50 days.

Between 2003 and 2011, a pilot study using combinations of human foetal tissue olfactory ensheathing cells, neural progenitor cells, umbilical cord mesenchymal cells, and Schwann cells was conducted. This trial consisted of 10 stroke patients (60 percent ischaemic stroke), 6 male and 4 female, aged between 42 to 87 years, 6 months to 20 years post-stroke. Cells were administered via several routes; intracranial parenchymal implantation, intrathecal implantation, or intravenous administration. Follow up was carried out 6 months to 2 years post-transplantation. The study demonstrated the clinical safety of this combination therapy, and every patient achieved improved neurological function (147).

In another clinical trial for ischaemic stroke patients, human foetal brain-derived neural stem/progenitor cells, and umbilical cord-derived MSC were intravenously administered (148). Neurological function and daily living abilities were improved, and no tumorigenesis was detected at 2 years. The study was small, but further provided evidence of safety and feasibility for cell combination therapy.

The first clinical trial to use NSC for chronic ischaemic stroke began in 2010, called the Pilot Investigation of Stem Cells in Stroke (PISCES) (149). This trial used a clonal cell line (ReNeuron's CTX0E03) of allogeneic human foetal brain-derived NSC genetically modified to express the immortalising gene, MYC. The expression is conditional and activated by tamoxifen, so proliferation and differentiation can be controlled. The phase I trial evaluated the safety of this cell line. Recruited patients were male, over the age of 60, 6 months to 5 years post-stroke, and with stable moderate to severe disability. They received a single dose of either 2, 5, 10, or 20 million cells in a single site (ipsilateral putamen), and data was collected over 2 years. No cell-related adverse events were observed up to the highest dose, and improved neurological function was exhibited. The trial involved a small number of patients, however it provided evidence of safety using this cell line and promising signs of efficacy. A phase II trial has now been started to assess safety and efficacy, and examine functional recovery of upper-limb movement. Patients enrolled include male and female, aged 40 and over, 3 months to 1 year post-stroke, with stable upper-limb disability. The dose of 20 million cells will be administered in a minimum cohort of 21 patients.

## Conclusion and Future Perspectives

Stem cell-based therapy is a promising alternative for stroke treatment. While stem cells from different sources, including induced PSC, ESC, MSC, and NSC, have been investigated, using NSC and enhancing the natural mechanisms is most appropriate for brain repair. In preclinical models of stroke, stem cell transplantation has led to positive outcomes through a variety of cellular and molecular mechanisms, many being mediated by the array of beneficial factors produced by the cells. Recent advances in cellular reprogramming have provided alternative sources of NSC to be investigated, allowing for safer and more efficient induction of NSC. More closely related cells, such as DPSC, are potential candidates for manipulation along the neural lineage. They have provided encouraging outcomes in stroke models and their neural replacement potential may be enhanced if resembling NSC. With more research, the limitations of cellular reprogramming can be overcome, and efficient, safe and reproducible methods for NSC induction can be developed.

## Author Contributions

MG: research and writing of manuscript. KK, SG, and SK: critical revision of manuscript for important intellectual content.

### Conflict of Interest Statement

The authors declare that the research was conducted in the absence of any commercial or financial relationships that could be construed as a potential conflict of interest.

## Abbreviations

BBB: blood-brain barrier
bFGF: basic fibroblast growth factor
BMSC: bone marrow stromal/stem cell(s)
CNS: central nervous system
DPSC: dental pulp stem cell(s)
EGF: epidermal growth factor
ESC: embryonic stem cell(s)
MSC: mesenchymal stem cell(s)
NSC: neural stem cell(s)
OCT4: octamer-binding transcription factor 4
PSC: pluripotent stem cell(s)
SDF1α: stromal cell-derived factor 1-alpha
SOX2: SRY (sex determining region Y)-box 2
VEGF: vascular endothelial growth factor.
